# The dangers of accepting a single diagnosis: case report of concurrent *Plasmodium knowlesi* malaria and dengue infection

**DOI:** 10.1186/s12936-016-1666-y

**Published:** 2017-01-03

**Authors:** Soon Eu Chong, Rhendra Hardy Mohamad Zaini, Siti Suraiya, Kok Tong Lee, Jo Anne Lim

**Affiliations:** 1 Department of Anaesthesiology and Intensive Care, School of Medical Sciences, Universiti Sains Malaysia, 16150 Kota Bharu, Kelantan Malaysia; 2 Advanced Medical and Dental Institute, Universiti Sains Malaysia, Bertam, 13200 Kepala Batas, Penang Malaysia; 3 Department of Medical Microbiology and Parasitology, School of Medical Sciences, Universiti Sains Malaysia, 16150 Kota Bharu, Kelantan Malaysia; 4 Hospital Raja Perempuan Zainab II, 15000 Kota Bharu, Kelantan Malaysia

**Keywords:** *Plasmodium knowlesi*, Dengue, Severe malaria, Co-infection, Malaysia, Acute respiratory distress syndrome

## Abstract

**Background:**

Dengue and malaria are two common, mosquito-borne infections, which may lead to mortality if not managed properly. Concurrent infections of dengue and malaria are rare due to the different habitats of its vectors and activities of different carrier mosquitoes. The first case reported was in 2005. Since then, several concurrent infections have been reported between the dengue virus (DENV) and the malaria protozoans, *Plasmodium falciparum* and *Plasmodium vivax.* Symptoms of each infection may be masked by a simultaneous second infection, resulting in late treatment and severe complications. *Plasmodium knowlesi* is also a common cause of malaria in Malaysia with one of the highest rates of mortality. This report is one of the earliest in literature of concomitant infection between DENV and *P. knowlesi* in which a delay in diagnosis had placed a patient in a life-threatening situation.

**Case presentation:**

A 59-year old man staying near the Belum-Temengor rainforest at the Malaysia–Thailand border was admitted with fever for 6 days, with respiratory distress. His non-structural protein 1 antigen and Anti-DENV Immunoglobulin M tests were positive. He was treated for severe dengue with compensated shock. Treating the dengue had so distracted the clinicians that a blood film for the malaria parasite was not done. Despite aggressive supportive treatment in the intensive care unit (ICU), the patient had unresolved acidosis as well as multi-organ failure involving respiratory, renal, liver, and haematological systems. It was due to the presentation of shivering in the ICU, that a blood film was done on the second day that revealed the presence of *P. knowlesi* with a parasite count of 520,000/μL. The patient was subsequently treated with artesunate-doxycycline and made a good recovery after nine days in ICU.

**Conclusions:**

This case contributes to the body of literature on co-infection between DENV and *P. knowlesi* and highlights the clinical consequences, which can be severe. Awareness should be raised among health-care workers on the possibility of dengue-malaria co-infection in this region. Further research is required to determine the real incidence and risk of co-infection in order to improve the management of acute febrile illness.

## Background

Dengue and malaria are two common mosquito-borne diseases in tropical countries, which are potentially fatal. Dengue is a viral disease transmitted by the ‘urban mosquito’ *Aedes aegypti*. The estimated global incidence of dengue is 390 million a year with about 96 million cases manifesting clinically [1]. Malaria is a protozoan disease transmitted by the ‘jungle mosquito’ *Anopheles* species. The World Health Organization (WHO) estimated that 214 million malaria infections occurred worldwide in 2015 and this has led to about 438,000 deaths [2].

Dengue fever can occur simultaneously with other virus, bacteria and protozoa infection [3–5]. Since the first reported case of dengue-malaria co-infection in France in 2005 [6], more cases have been reported in India [7, 8], Pakistan [9, 10], Brazil [11], East Timor [12], Bangladesh [13], Indonesia [14], Cambodia [15], Malaysia [16], and Japan [17]. Among these reported cases, only *Plasmodium falciparum* and *Plasmodium vivax* were involved. This case involved a concurrent infection of dengue virus (DENV) and *Plasmodium knowlesi,* which had misled clinicians resulting in delayed treatment with life-threatening consequences.

## Case report

The patient was a 59-year-old man from the town of Jeli in Malaysia. Jeli lies on the edge of the Belum-Temengor rainforest, one of Malaysia’s national reserves bordering Thailand. Gradual urbanization over the past few decades has made this town an outbreak area of dengue and leptospirosis.

The patient was admitted with fever, headache, myalgia, arthralgia, and poor oral intake over the 6 days prior to admission. He was given antipyretic and amoxicillin by his general practitioner, but his condition had worsened. No blood investigation was done. On the day of presentation, he had difficulty in breathing and was referred to hospital.

At the emergency department, the patient was conscious but lethargic. He was mildly dehydrated, blood pressure (BP) was 102/78 mmHg and heart rate was 104 bpm. Capillary refill time was less than 2 s with a high, spiking temperature of 40 °C. He was mildly jaundiced and abdomen was soft with no organomegaly. There were no signs of haemorrhagic manifestation. Respiratory rate was 35 breaths/min and oxygen saturation (SPO_2_) was 93% on ambient air. Lungs were clear and breath sounds were equal bilaterally. He had type I respiratory failure, with arterial blood gas (ABG) pH of 7.36, PaCO_2_ at 25.6 mm Hg, PaO_2_ at 77.4 mm Hg, bicarbonate at 21.2 mmol/L, and lactate at 1.8 mmol/L on face mask oxygen of 5 L/min. Non-invasive ventilatory (NIV) support was required to improve oxygenation. Bedside abdominal ultrasonography performed showed no hepatosplenomegaly with a normal biliary tract. Inferior vena cava (IVC) was collapsed at 0.88 cm, with a collapsibility index of more than 50%. Fluid challenge with normal saline was initiated and titrated up to 10 mL/kg/h.

Investigations done in the emergency department revealed a platelet count of 33 × 10^9^/L and a haematocrit (HCT) level of 39.4%. A rapid test using immunochromatic technique (ICT) was positive for dengue non-structural protein1 (NS1) antigen; similarly, the serologic Anti-DENV Immunoglobulin M (IgM) testing. International normalized ratio (INR) was 1.23 and aPTT was 44.3 s. Other laboratory investigation results included: haemoglobin (14.3 g/dL), red blood cell (RBC) count (4.55 × 10^12^/L) and white blood cell (WBC) count (6.91 × 10^9^/L). Hyponatraemia (131 mmol/L) was present and liver function test was deranged with a total bilirubin of 46 mmol/L, alanine transferase of 77 mmol/L and alkaline phosphatase of 151 mmol/L (Table 1). A blood film for malaria parasites was not carried out.

**Table 1:** Laboratory findings and progress

Investigations	Normal range	Day 1 (of admission)	Day 2	Day 3	Day 4	Day 6	Day 9
Parasite counts (/µL)		–	520,000	460,000	320	120	Nil
Haemoglobin (g/dL)	[13.5–17.5]	14.3	11.8	11.4	8.8	10.2	9.1
Platelet count (×10^9^/L)	[150–450]	33	17	20	78	175	277
Haematocrit (%)	[42–54]	39.4	32.8	32.1	24.4	28.2	26.5
White blood cell count (×10^9^/L)	[4000–11,000]	6.91	6.87	9.21	10.97	17.39	9.25
Red blood cells (×10^12^/L)	[4, 5]	4.55	3.78	3.72	2.95	3.44	3.15
Sodium (mmol/L)	[135–150]	131	137	135	134	137	139
Potassium (mmol/L)	[3.5–5.0]	4.5	5.4	5.3	4.7	4.1	3.9
Urea (mmol/L)	[1.0–8.3]	7.9	18.2	29.7	26.4	29.8	19.6
Creatinine (µmol/L)	[70–120]	116	275	405	425	512	346
Total bilirubin (mmol/L)	[3–17]	46	44	39	35	25	17
Aspartate aminotransferase (U/L)	[12–37]	80	104	92	78	37	31
Alanine aminotransferase (U/L)	[20–65]	77	70	50	33	31	23
Alkaline phosphatase (U/L)	[50–136]	151	135	119	97	110	95
Lactate dehydrogenase (U/L)	[140–280]	994		2164		1680	
Creatine kinase (U/L)	[<170]	177		221			
INR	[0.8–1.2]	1.23	1.46	1.42	1.21	1.39	1.34
Prothrombin time (s)	[11–14]	15.4	17.5	17.2	15.2	16.9	16.4
aPTT (s)	[25–35]	44.3	55.9	51.0	54.8	59.9	47.6
NS1 antigen		Positive					
Dengue IgM		Positive					
CRP (mg/L)	[<1]	>200		>200		>200	
Random blood sugar (mmol/L)	[6–10]	6.5	5.0	5.7	6.1	5.3	5.2
ABG		FMO_2_ 5 L/min	CPAP FiO_2_ 60%	BIPAP FiO_2_ 60%	BIPAP FiO_2_ 60%	CPAP FiO_2_ 40%	NPO_2_ 3 L/min
pH	[7.5–7.45]	7.36	7.247	7.28	7.35	7.43	7.43
PaO_2_ (mmHg)	[>80]	77.4	100	133	142	89.1	111
PaCO_2_ (mmHg)	[35–45]	25.6	30.4	25.7	28.8	30.9	38
HCO_3_ (mmol/L)	[22–26]	21.2	16.1	14.3	17.7	22	25.5
Lactate (mmol/L)	[<2]	1.8	3.5	2.8	1.2	0.8	0.6

*ABG* arterial blood gas, *aPTT* activated partial thromboplastin time, *BIPAP* bilevel positive airway pressure, *CPAP* continuous positive airway pressure, *CRP* C-reactive protein, *FMO2* face mask oxygen, *IgM* immunoglobulin M, *INR* international normalized ratio, *NS1* nonstructural protein 1, *PaCO*_*2*_ partial pressure of carbon dioxide, *PaO*_*2*_ partial pressure of oxygen

The patient was diagnosed as a severe dengue case and admitted to the intensive care unit (ICU) for closer observation. Bedside echocardiography revealed a normal cardiac function and IVC had picked up to 1.9 cm with the collapsibility index reduced to less than 50%, which corresponded to an improved volume status. However, the serum lactate level was still increasing, acidosis worsening and urine output decreasing. Further fluid challenge was administered carefully up to 50 mL/kg over 8 h with regular IVC assessment. Intravenous ceftriaxone, 2 g daily, was initiated by the physician to cover the possibility of concomitant leptospirosis because the patient was from an outbreak area.

**Fig. 1:**
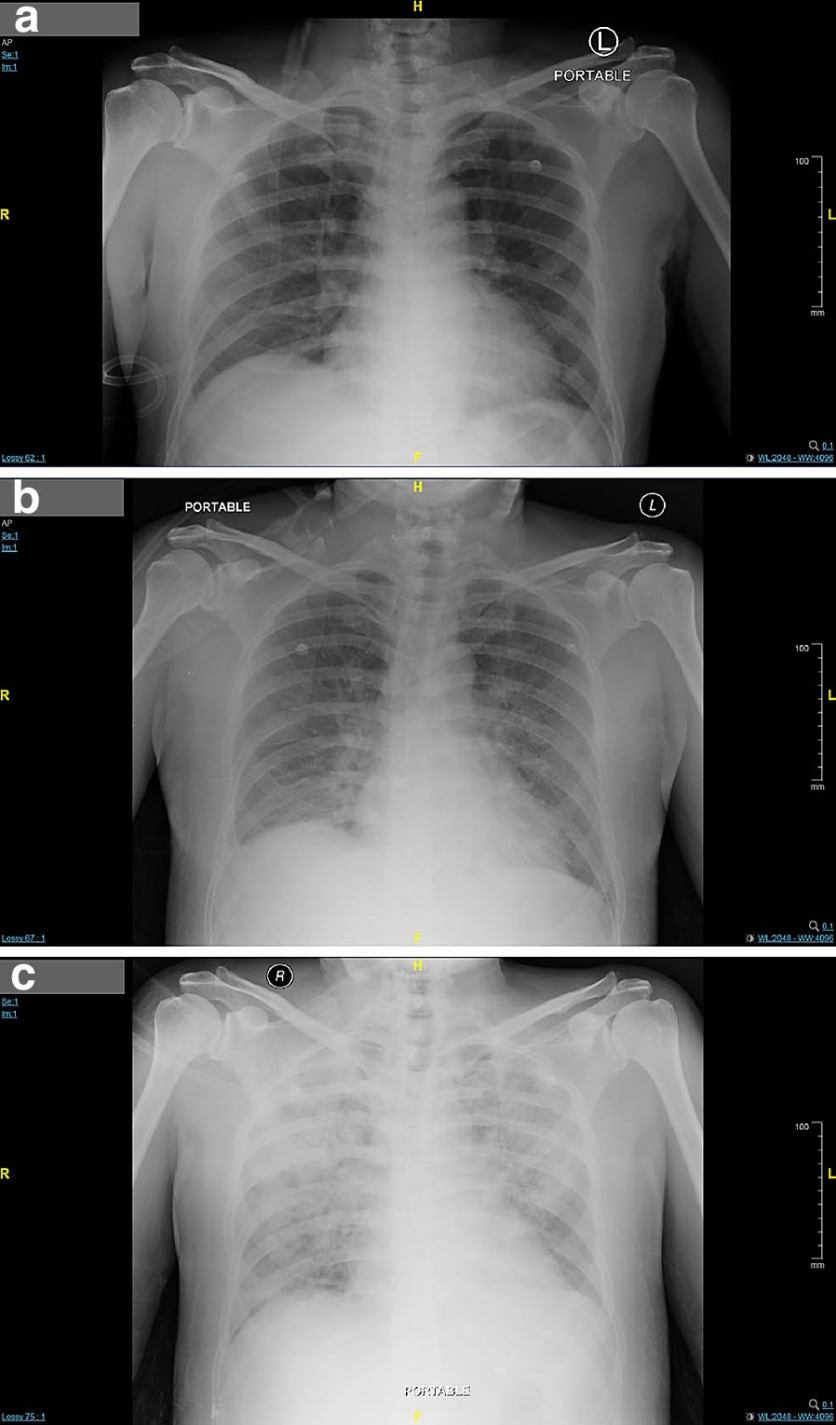
**a** Portable chest radiograph (AP view) looks normal on presentation to the emergency department (day 1); **b** chest radiograph repeated the following day showed the presence of perihilar haziness and increased pulmonary vascular markings (day 2); **c** chest radiograph showed increased air-space opacity in both lungs and parapneumonic effusion (day 6). *AP* anteroposterior

On the following day, the patient’s oxygenation deteriorated and NIV support had to be increased gradually. Under an FiO_2_ of 60%, ABG showed: pH 7.247, pCO_2_ 30.4 mmHg, pO_2_ 100 mmHg, HCO_3_ 16.1 mmol/L and lactate 3.5 mmol/L. A chest radiograph repeated the following day showed the presence of perihilar haziness and increased pulmonary vascular markings (Fig. 1b). The patient’s platelet counts dropped further to 17 × 10^9^/L but there was no sign of spontaneous bleeding. He also went through a few hypotensive episodes where the BP dropped to 85/50 mmHg and mean arterial pressure down to 60 mmHg. Vasopressor had to be initiated to maintain a mean arterial pressure of above 65 mmHg. Meanwhile, he suffered acute kidney injury with a urine output of 5–10 mL/h, requiring haemodialysis. Although it was the seventh day of illness (second day from admission), the patient had a spiking temperature of 38.7 °C. The fever was preceded by severe shivering which lasted for nearly an hour. This shivering led clinicians to perform a blood film for malaria parasite (BFMP). The result showed the presence of the malaria parasite resembling *Plasmodium knowlesi* with parasitaemia of 520,000/µL of blood. Intravenous artesunate 2.4 mg/kg daily and doxycycline 100 mg twice a day were started. Subsequently, polymerase chain reaction (PCR) test confirmed the parasite to be *P. knowlesi*. Soon after starting anti-malarial treatment, the malaria parasite count dropped to 460,000/μL after 24 h and 320/μL after 48 h, respectively. The patient’s platelet count started to improve as well (Table 1).

On the sixth day from admission, the patient’s respiratory symptoms worsened again with coarse crackles heard over both lungs on auscultation. He required NIV support to maintain an SPO_2_ of more than 92%. From ABG, PaO_2_ was 80 mmHg with FiO_2_ of 60%. Chest radiography showed increased air-space opacity in both lungs and parapneumonic effusion (Fig. 1c). His WBC count raised to 17.4 × 10^9^/L with predominant neutrophilia, and C-reactive protein was more than 200 mg/L. He was then treated for hospital-acquired pneumonia with acute respiratory distress syndrome (ARDS). Intravenous meropenam was initiated.

**Fig. 2:**
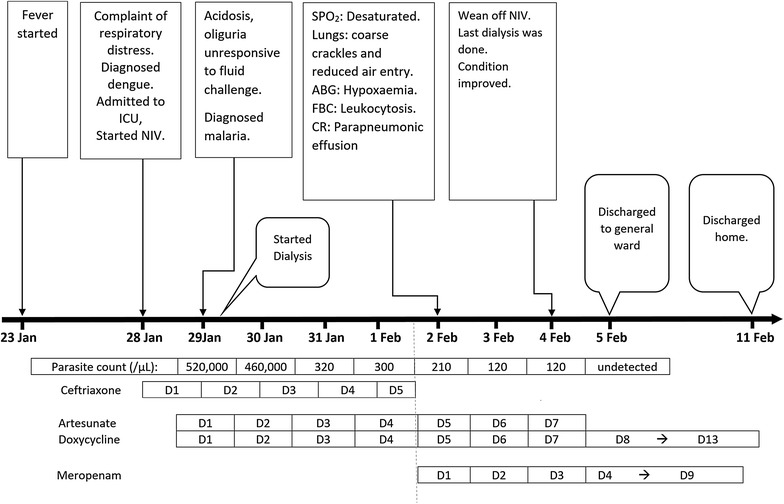
Timeline of key events. Timeline in the diagnosis, treatment and progress of the patient with concomitant dengue and *Plasmodium knowlesi* malaria (*upper part*). Progress of parasite count and usage of antibiotics (*lower part*). *ABG* arterial blood gas, *CR* chest radiography *FBC* full blood count, *ICU* intensive care unit, *NIV* non-invasive ventilation, *SPO*_*2*_ oxygen saturation

With close monitoring and vigilant treatment, the patient’s condition improved. His oxygenation, renal function, liver function, and platelet count returned to normal. He was successfully weaned off NIV support on the eighth day from admission. Blood and urine cultures all yielded negative results. Serology for leptospirosis, syphilis, hepatitis B, hepatitis C and HIV screenings were negative. Parasites were cleared from his blood on the eighth day after starting artesunate and doxycycline. He was finally able to be transferred to the general ward, and was discharged on the 15th day of hospitalization (refer Fig. 2 for event timeline). On subsequent follow-up the following month, he was discharged from the medical clinic without serious complications.

## Discussion

The first case of dengue-malaria dual infection was reported in France in 2005 [6] in which *P. falciparum*-DENV co-infection was detected, followed by a few more case reports. One year later in 2006, cases on *P. vivax*-DENV dual infection were reported [18, 19]. Although concurrent infection is uncommon, it has subsequently been mentioned with increasing frequency in literature. There were isolated case reports in India [7, 20–24], East Timor [12], Japan [17], Bangladesh [13], Indonesia [14], Malaysia [16], and Cambodia [15]. Following that, a series of cases were reported in French Guiana [25, 26], Brazil [11, 27], India [28–30], Bangladesh [31], Pakistan [10], Peru [32], and Kenya [33].

Studies were conducted regionally and it was noticed that the incidence of dual malaria-dengue co-infection among patients presented with acute febrile illness was actually higher than expected, ranging from 1 to 33% of the acute febrile illness state in different countries [8–10, 26, 33]. This increase may be explained by the overlap of vectors in endemic areas and increased prevalence of dengue fever [9, 26].

Thailand has been one of the dengue and malaria outbreak regions in Southeast Asia [2, 34]. It is an important country in malaria research because there have been reports on the artemisinin-resistant strain of falciparum malaria at the Thai–Cambodian border and the Greater Mekong Sub-region (Cambodia, Laos, Myanmar, Viet Nam) [2, 35, 36]. Surprisingly, in a study conducted in Thailand in 2006 by Singhsilarak et al. involving 194 patients with malaria, no co-infection with dengue was found [37]. To date, there has been only one case reported in Thailand on dual dengue-malaria infection involving *P. falciparum*-DENV [38]. With this current incidence occurring near the Malaysia–Thailand border, awareness among doctors in this region is crucial with regard to the possibility of co-infection. Both diseases are endemic in Southeast Asia, especially in areas near to jungle that is undergoing urbanization. As for Malaysia, both diseases may overlap in Sarawak and Sabah [39], Pahang and Kelantan [40].

Clinical presentations of *P. falciparum*-DENV co-infections have been described over the past decade [6, 12, 17], as well as that of *P. vivax* and DENV co-infection [10, 11, 18]. This is probably one of the first few reports of concurrent *P. knowlesi*-DENV dual infection. There have been two cases of *P. knowlesi*-DENV co-infection reported before [41, 42], but this article is the first with a detailed case scenario and description of the dangers if misdiagnosed.

*Plasmodium knowlesi* is one of the most common causes of malaria-related deaths in Malaysia [2, 43]. It was first detected in Malaysia in 1965 but faced diagnostic difficulty because of its similar morphology with *Plasmodium malariae*. Unfortunately, it gives a more severe clinical manifestation compared to *P. malariae*. It can be associated with high parasitaemia and, like *P. falciparum,* it can be fatal [41, 43, 44]. In view of the diagnostic difficulty and the high mortality risk, the Malaysian Malaria Management Guidelines recommend that all blood film results with parasites resembling *P. malariae* should be reported as *P. knowlesi/P. malariae* and patients should be treated as *P. knowlesi* infection [45].

Knowlesi malaria is a simian malaria which is unlike other types of malaria as it can be carried by Old World monkeys [46, 47]. The eradication of this parasitic disease is expected to be difficult and not achievable in the near future. The incidence of co-infection should be taken seriously. Reported cases of concurrent infection in Southeast Asia are far too few compared to the dengue-malaria co-infection rate, from 1 to 33%: in French Guiana (1%) [26], Kenya (5.7%) [33], India (10.25%) [8], and Pakistan (23.21–33%) [9, 10] in local studies. The actual number of incidences are probably underestimated as such areas are known to be highly endemic for both malaria and dengue, especially around the forest area of Myanmar, Thailand, Laos, Vietnam, East Timor, Papua, Sabah, and Sarawak.

Incidence of co-infection with dengue and malaria is likely to increase as today’s population is highly mobile, with increased activities brought about by good transportation systems. These two diseases share similar major symptoms such as fever, headache, myalgia, arthralgia, rash, nausea, diarrhoea, vomiting, and abdominal pain. Differentiating them is difficult on clinical grounds alone. One of the clinical signs that may suggest malaria rather than dengue in this context will be splenomegaly [48]. However, this patient had no splenomegaly and absence of splenomegaly does not rule out malaria. With the emergence of malaria-dengue co-infections, even with laboratory investigations, the risk of overlooking malaria is still high in dengue outbreak areas and vice versa. This is because they both share similar laboratory findings with dengue, such as thrombocytopaenia. In Malaysia, the confirmation tests for dengue (NS1 antigen and dengue IgM) are rapid and easily available. A positive confirmatory dengue test often misleads practitioners to rule out the possibilities of other diseases without actually screening against it [42, 49, 50].

The challenges for a reliable clinical diagnosis are further increased with atypical manifestations of dengue fever in recent years. These include dengue encephalitis, dengue myocarditis, dengue with ARDS and pleural effusion, dengue-induced hepatitis, and severe dengue causing kidney failure [51]. However, there are some signs for clinicians to suspect the presence of concomitant infection with dengue. Firstly, in most cases of dengue, the fever spikes by day 5 of illness. Fever may be prolonged in patients who have co-existing infections [52]. Secondly, patients with dengue are more likely to have a temperature under 39 °C [48]. Thirdly, they are more likely to be associated with leucopenia [48], haemo concentration [8] and atypical lymphocytosis [34, 35]. In this patient, the temperature was 40 °C on day 6 of illness, there was no haemoconcentration and no leucopenia. Additionally, he had anuria, worsening metabolic acidosis and hyperlactatemia, which was resistant to aggressive fluid resuscitation. These are strong indications to further investigate co-existing infections. In fact, a leptospirosis test was taken on admission with prophylactic treatment for leptospirosis. Unfortunately, BFMP was not done upon admission.

There is a scoring system based on clinical and laboratory criteria, described by Epelboin et al. [52], to predict the need for a parasitological examination in a febrile patient with atypical symptoms (Table 2). The patient scored 13/14 in this scoring upon admission and was indicated for a BFMP screening.

**Table 2:** Scoring to differentiate dengue monoinfection and dengue-malaria coinfection

Criteria towards dengue	Score
Male gender	1
C-reactive protein >5 mg/L	9
Age >15 years old	1
Platelet count <100.10^9^/L	2
Haematocrit <36%	1

Criteria fulfilled by the patient

1. Score <10: low risk of malaria

2. Score ≥10: Indication for parasite examination

Sensitivity of 0.997 (95% CI 0.995–1); specificity of 0.41 (95% CI 0.32–0.50)

Cited from: Epelboin et al. [52]

The most important lesson from this case report was that the delay in suspecting malaria infection caused a delay in treatment, which resulted in the deterioration of the patient’s condition. The history that the patient’s home was near the jungle should have prompted an early BFMP test but it was only when he developed uncorrected acidosis and the presence of shivering that this was done.

A timely diagnosis of malaria is of utmost importance because with early treatment the outcome is good. If left untreated, this condition can rapidly turn fatal as *P. knowlesi* has a 24-h replication cycle [53] (48 h for *P. falciparum*, *P. vivax* and *P. ovale*; 72 h for *P. malariae* [54]) which causes a drastic rise in parasitaemia leading to multiple organ failure. Additionally, treatment of these two illnesses differs. Most dengue cases can be treated with adequate supportive management, i.e., fluid replacement and other measures. Supportive management itself may reduce the mortality rate from more than 20% to less than 1% in severe dengue cases [55]. To treat malaria, specific anti-malarial medication has to be used or the infection may rapidly become fatal [56, 57]. In a case reported in East Timor, a girl who falsely tested negative for malaria with a rapid diagnostic kit died within 7 h of admission because of delayed treatment [12]. There were several mortalities in Malaysia where parenteral artesunate was delayed because *P. knowlesi* was wrongly reported as *P. malariae*, which has a relatively benign clinical course [57]. Awareness and a high index of suspicion of the possibility of dengue-malaria co-infection can be life-saving and any suspicion of such case must be carefully excluded.

The severe manifestation of this patient may be multifactorial. Apart from the delay in diagnosis and treatment, it can also be due to the co-infection of malaria and dengue which caused an exaggerated host immune response, as in the theory proposed by Mendonca et al. [58]. Concurrent infections have also been suggested to be more severe than isolated infection [25, 58]. However, this statement remains controversial. Halsey et al. reported no difference in severity between isolated infection and co-infection [32].

## Conclusion

This article aims to contribute to the body of knowledge on the presentation, progress and management of *P. knowlesi*-DENV co-infection. This may aid similar encounters in future, especially in Southeast Asia where *P. knowlesi* abounds. The medical fraternity should be alert regarding the possibility of co-existing infection which may mimic the atypical presentations of dengue fever. Dengue mortality in Malaysia has been increasing [59], any emergence of undiagnosed concomitant malaria may worsen this situation. A high index of suspicion is of utmost importance to prevent deadly complications arising from delayed treatment. Further research is required to determine the real incidence in order to guide the management of acute febrile illness.

## Abbreviations

ABG: arterial blood gas
ARDS: acute respiratory distress syndrome
BFMP: blood film for malaria parasite
BP: blood pressure
bpm: beats per minute
DENV: dengue virus
FiO_2_: fraction of inspired oxygen
HCO_3_: bicarbonate level
HCT: haematocrit
ICT: immunochromatic technique
ICU: intensive care unit
Ig M: immunoglobulin M
IVC: inferior vena cava
NIV: non-invasive ventilation
NS1: non-structural protein 1
PaO_2_: partial pressure of arterial oxygen
PaCO_2_: partial pressure of arterial carbon dioxide
PCR: polymerase chain reaction
RBC: red blood cells
SBP: systolic blood pressure
SPO_2_: oxygen saturation
WBC: white blood cell
WHO: World Health Organization
